# Transcriptomic comparison of *Aspergillus niger *growing on two different sugars reveals coordinated regulation of the secretory pathway

**DOI:** 10.1186/1471-2164-10-44

**Published:** 2009-01-23

**Authors:** Thomas R Jørgensen, Theo Goosen, Cees AMJJ van den Hondel, Arthur FJ Ram, Jens JL Iversen

**Affiliations:** 1 Department of Biochemistry and Molecular Biology, University of Southern Denmark, Campusvej 55, 5230 Odense M, Denmark; 2 Institute for Biology, Leiden University, Kluyver Centre for Genomics of Industrial Fermentation, Wassenaarseweg 64, 2333 AL Leiden, the Netherlands; 3 BioCentre, HAN University, Laan van Scheut 2, 6525 EM Nijmegen, the Netherlands

## Abstract

**Background:**

The filamentous fungus, *Aspergillus niger*, responds to nutrient availability by modulating secretion of various substrate degrading hydrolases. This ability has made it an important organism in industrial production of secreted glycoproteins. The recent publication of the *A. niger *genome sequence and availability of microarrays allow high resolution studies of transcriptional regulation of basal cellular processes, like those of glycoprotein synthesis and secretion. It is known that the activities of certain secretory pathway enzymes involved *N*-glycosylation are elevated in response to carbon source induced secretion of the glycoprotein glucoamylase. We have investigated whether carbon source dependent enhancement of protein secretion can lead to upregulation of secretory pathway elements extending beyond those involved in *N*-glycosylation.

**Results:**

This study compares the physiology and transcriptome of *A. niger *growing at the same specific growth rate (0.16 h^-1^) on xylose or maltose in carbon-limited chemostat cultures. Transcription profiles were obtained using Affymetrix GeneChip analysis of six replicate cultures for each of the two growth-limiting carbon sources. The production rate of extracellular proteins per gram dry mycelium was about three times higher on maltose compared to xylose. The defined culture conditions resulted in high reproducibility, discriminating even low-fold differences in transcription, which is characteristic of genes encoding basal cellular functions. This included elements in the secretory pathway and central metabolic pathways. Increased protein secretion on maltose was accompanied by induced transcription of > 90 genes related to protein secretion. The upregulated genes encode key elements in protein translocation to the endoplasmic reticulum (ER), folding, *N*-glycosylation, quality control, and vesicle packaging and transport between ER and Golgi. The induction effect of maltose resembles the unfolded protein response (UPR), which results from ER-stress and has previously been defined by treatment with chemicals interfering with folding of glycoproteins or by expression of heterologous proteins.

**Conclusion:**

We show that upregulation of secretory pathway genes also occurs in conditions inducing secretion of endogenous glycoproteins – representing a more normal physiological state. Transcriptional regulation of protein synthesis and secretory pathway genes may thus reflect a general mechanism for modulation of secretion capacity in response to the conditional need for extracellular enzymes.

## Background

The black-spored mitosporic fungus, *Aspergillus niger*, is specialized to grow on plant cell wall- and storage-polysaccharides such as xylans, pectins, starch and inulin [1,2]. It does so by secreting high levels of a wide range of substrate degrading enzymes into its habitat. Enzyme mediated degradation of plant polysaccharides results in liberation of monomeric carbohydrates, which are efficiently taken up and metabolised by the fungus. The inherent high enzyme secretion capacity of *A. niger *and its high productivity of organic acids, like citric acid, has made it an interesting organism to study processes such as protein production and primary metabolism [3,4]. Members of the genus *Aspergillus*, including *A. niger*, are also reputed for biosynthetic potential of a variety of mycotoxins [5], such as the carcinogenic aflatoxins [6,7] and ochratoxins [8] and, as discovered recently in *A. niger*, also the carcinogenic fumonisins [3,9].

In eukaryotic cells, protein secretion involves ER-associated translation, folding and modification of proteins, which are then transported via vesicles to the Golgi apparatus or other compartments for further modification. The mature glycoproteins are finally transported with secretory vesicles to the cell membrane and secreted into the environment. The components and mechanisms of the secretory pathway in eukaryotes are highly conserved. Main elements of the secretory pathway in fungi and mammals are described in recent reviews [10-13]. A genomic comparison of genes encoding secretory pathway components in *A. niger*, *Saccharomyces cerevisiae *and mammals has not revealed major differences in the number of genes involved in protein secretion and the analysis did not explain why *A. niger *is a more efficient secretor of extracellular proteins than the yeast *S. cerevisiae *[3]. However, it has been shown that activity of certain secretory pathway enzymes involved *N*-glycosylation is elevated in response to overexpression of the glycoprotein glucoamylase in *A. niger *[14]. There is also a positive correlation between glucoamylase expression and activity of glycosylation enzymes when comparing growth on maltodextrin, which induces glucoamylase expression, to growth on xylose, which is a non-inducing carbon source [14]. These observations suggest that *A. niger *can adapt the activity of at least parts of its secretory pathway to handle the increased load of secreted proteins induced by a given environment. In the present work, we have investigated whether carbon source dependent enhancement of protein secretion can lead to upregulation of secretory pathway elements, which extend beyond those involved in *N*-glycosylation.

Consequently, we have compared transcriptomic profiles of *A. niger *cultures, expressing the endogenous glucoamylase gene, growing on a glucoamylase-inducing carbon source, maltose, to profiles from cultures growing on xylose (non-inducing). We have used carbon-limited chemostat cultivation to control the specific growth rate (μ) during growth on the two different carbon sources and to obtain highest reproducibility in well defined culture conditions.

We show that the rate of protein secretion is 2–3 times higher on maltose compared to xylose, and that the increased protein secretion by *A. niger *on maltose is accompanied by upregulation of transcription of more than 90 genes encoding elements of the secretory pathway. Most of the upregulated secretory pathway elements reside in the ER or are involved in vesicle trafficking between ER and Golgi. The transcriptional response to maltose resembled the unfolded protein response (UPR) induced by diverse types of artificial ER-stress [15]. We suggest that the transcriptional regulation of the secretory pathway is part of a physiological mechanism, which has evolved to allow varying output of substrate degrading enzymes.

## Results and discussion

### Physiology of xylose- or maltose-limited chemostat cultures of A. niger

**Figure 1:**
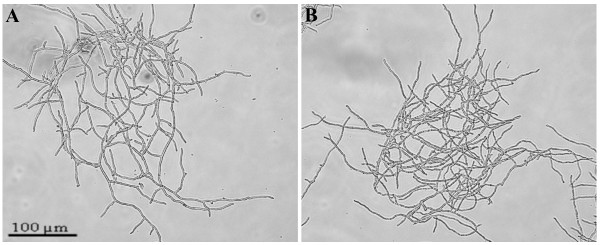
**Morphology of mycelium in chemostat cultures of *A. niger***. (A) Steady state on xylose (50 h). (B) Steady state on maltose (80 h).

Steady state cultures, growing on xylose or maltose, were homogenous and characterized by dispersed filamentous hyphae (Fig. 1), and during the whole cultivation only minimal amount of biomass adhered to surfaces in the reactor (< 0.5 g dry biomass in the end). Carbon was accounted for in carbon balances of the influent medium and effluent culture broth and exhaust gas. The carbon-recoveries were approximately 100% (Table 1), thus validating that μ was equal to the dilution rate (D = 0.16 h^-1^) in all 12 steady state cultures. In addition, the relatively large volume (4.3 l) and steady-state biomass concentration of the cultures allowed sampling of sufficient material for analyses without perturbations of the steady state.

**Table 1:** Physiology in chemostat culture.

**C-source**	**Strain**	**C**_**biomass**_ (g_DW _kg^-1^)	**C**_**xylose/maltose **_^†^ (μM)	**Y**_**x/s**_ (g_DW _g_substrate_^-1^)	**Y**_x/C_ (g_**DW**_g_carbon_^-1^)	**q**_**CO2**_ (mmol g^-1 ^h^-1^)	**q**_**O2**_ (mmol g^-1^h^-1^)	**RQ**	**q**_**protein-EC**_ (mg g^-1 ^h^-1^)	**C-recovery** (%)
Xylose	AB94-85	4.06 ± 0.07	233 ± 6	0.54 ± 0.02	1.35 ± 0.04	3.40 ± 0.11	3.36 ± 0.14	1.01 ± 0.02	0.68 ± 0.01	98 ± 2
	ABGT1026	3.96 ± 0.07	256 ± 18	0.53 ± 0.01	1.32 ± 0.03	3.41 ± 0.10	3.42 ± 0.10	1.00 ± 0.01	0.72 ± 0.02	101 ± 3
Maltose	AB94-85	3.68 ± 0.08*	160 ± 5	0.52 ± 0.01	1.23 ± 0.03*	2.92 ± 0.05*	3.38 ± 0.14	0.87 ± 0.05*	1.98 ± 0.28*	101 ± 1
	ABGT1026	3.68 ± 0.12*	158 ± 24	0.52 ± 0.02	1.23 ± 0.04*	2.84 ± 0.17*	3.57 ± 0.27	0.80 ± 0.10*	1.69 ± 0.24*	97 ± 2

Steady state results of chemostat cultures with xylose or maltose as growth-limiting substrates. Standard deviations ( ± ) are given for mean values of triplicate independent steady state results. C_biomass_, dryweight biomass concentration; C_xylose/maltose_, concentration of substrate (NB: maltose as glucose equivalents); Y_xs_, growth yield on substrate; Y_xC_, growth yield on substrate carbon; q_CO2_, specific carbon dioxide evolution rate; q_O2_, specific oxygen consumption rate; RQ, respiratory quotient calculated as the ratio of C_O2 _production and O_2 _consumption rates; q_protein-EC_, specific production rate of extracellular protein; C-recovery, carbon recovery. Two-tailed t-tests were used to evaluate significance (p < 0.05) of differences in each column (except substrate concentration and carbon recovery).

* results are significantly different from unmarked results in the same column.

† substrate concentration on maltose is given as concentration of glucose released into filtrate after incubation with α-glucosidase.

**Figure 2:**
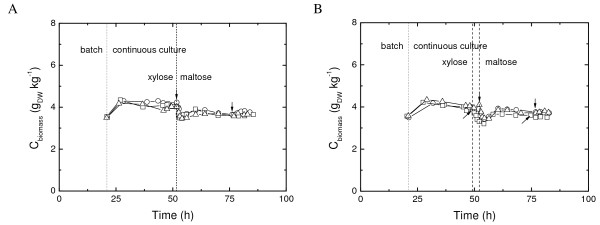
**Growth profiles of triplicate *A. niger *AB94-85 (A) and ABGT1026 (B) chemostat cultures**, Dry weight biomass concentration (gDW kg^-1^) as a function of time (h) illustrates growth of three replicate cultures (open square, circle and triangle). Dot-line indicates start of continuous cultivation – exit from batch culture. Dash-line represents the switch to maltose as carbon source after 5 RT with xylose as the growth-limiting substrate. Arrows indicate time-points, where mycelium was harvested for transcriptomic analysis.

Growth physiology of two *A. niger *strains was evaluated from triplicate chemostat cultures. The duration of initial xylose-grown batch cultures was approximately 21 h, followed by approximately 63 h of continuous cultivation. After five volume changes (5 × D^-1^) of xylose-limited growth, the xylose-containing growth medium was replaced with a medium containing maltose. Growth was then followed for another five residence times. As shown in Fig. 2, the shift of carbon source led to a transient decrease of the biomass concentration, indicating that the cells were unable to grow at the same specific growth rate on maltose during the first hour after the shift. Once the metabolic machinery necessary for consumption of maltose was induced, growth rate increased and approximately eight hours after the shift the biomass concentration stabilized at a new steady state value. RNA for transcriptome analysis was isolated from steady state cultures on xylose or maltose after at least four residence times on each carbon source. Growth profiles and sampling events are shown in Fig. 2.

The results listed in Table 1 summarize steady state physiology of xylose- and maltose-limited chemostat cultures. The reproducibility of the triplicate cultures was very high. The coefficient of variation (CV) of steady state biomass concentrations was approximately 0.02 for both carbon sources. While replicate variability was low, there were marked differences between cultures grown on xylose or maltose. Notably, specific productivity of extracellular protein (q_protein-EC_) was 2 to 3 fold higher on maltose than on xylose, indicating increased protein-secretory activity of maltose-limited cultures. The biomass yield on carbon (Y_xC_) was lower on maltose, probably at the expense of increased product formation. The carbon concentration in the culture filtrate and the acidification rate of maltose-limited cultures were higher compared to xylose-limited cultures (results not shown). These observations, taken together with a respiratory quotient (RQ) lower than 1 of maltose-limited cultures, most likely reflect higher productivity of organic acids in addition to increased protein secretion on maltose.

### Reproducibility of steady state gene-expression

**Figure 3:**
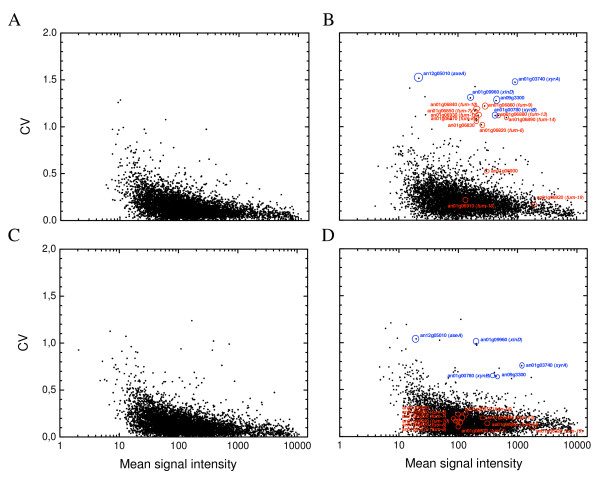
**Signal intensity variation among replicate cultures**. Variation is expressed as coefficient of variation (CV) of mean signal intensities of independent triplicate measurements, and shown for steady-state gene-expression of xylose- (A, C) and maltose-limited (B, D) cultures of AB94-85 and ABGT1026. Only genes with Detection call Marginal or Present in at least one of three measurements are shown (expressed genes). Blue and red circles and identifiers indicate maltose-expressed genes involved in xylose/xylan catabolism and clustered fumonisin biosynthesis gene homologs, respectively.

Microarray analysis revealed, that less than 50% of the 14,165 predicted open reading frames (ORF) were transcribed on any of the two substrates, xylose or maltose (raw signal intensities and detection calls for all genes are given in [Additional file 1]). The high reproducibility of replicate chemostat cultures was reflected by a very low degree of replicate variation in transcript levels (Fig. 3). The average CV of all genes expressed was 0.14–0.15 and 0.17–0.22 in triplicate xylose- and maltose-limited chemostat cultures, respectively. This is fully comparable to the level of reproducibility reported for genome-wide transcription in chemostat cultures of *S. cerevisiae *[16]. A closer look at some (14) of the genes with most variable transcript level on maltose, reveals interesting correlations (Fig. 3B, D). Five of the genes are involved in metabolism/degradation of xylose or xylans and had varying transcript level in replicate cultures. Transcription of xylose-related genes, in absence of xylose, indicates derepression or influence of previous growth conditions under xylose-limitation, where all five genes were highly expressed. However, after 25 h or 4 residence times (RT) of continuous cultivation with maltose, more than 98% of the culture from xylose-limited steady state had been removed and any residual xylose depleted.

Nine other genes with a CV higher than 1 were all more expressed in a replicate culture of AB94-85 (culture "#95") on maltose. These genes are all part of the putative fumonisin gene cluster in *A. niger *[3]. In total, twelve genes in the fumonisin gene homolog cluster were expressed on maltose [see Additional file 2]. This appears to correspond to all homologous genes needed for biosynthesis of fumonisins in *Fusarium verticillioides*. Recently, Frisvad *et al*. [9] reported of fumonisin B2 production by progenitors of three genome sequenced *A. niger *strains, including the source of the strains in this study (*A. niger *N400). The variable transcription of homologs in a putative mycotoxin gene cluster demonstrates the need for careful evaluation of a given strain s mycotoxin-producing ability.

### Carbon source dependent gene expression

Analysis of variance (Two-Way ANOVA) identified approximately 1,250 genes differentially expressed (significance: p < 0.005) in response to the carbon source, xylose or maltose, and independent of strain background [see Additional file 3]. Transcription of 57% of these were higher on maltose compared to xylose in steady state cultures. Most changes in gene expression were characterized as low-fold differences; for expression of about 890 genes the difference was less than 2-fold. Low-fold differences (< 2) are often precluded from studies due to limitations set by sample size and reproducibility. Generally, one would expect that differences in transcript levels of genes encoding basal functions and elements of basal pathways consisting of many interdependent processes will be small. Transcription of genes encoding functions specific to one condition display high-fold differences, as evidenced by genes involved in conversion of the sole carbon source, xylose, to the pentose phosphate pathway intermediate, xylulose-5-phosphate (Table 2), whereas central metabolic processes, also needed in the other condition (growth on maltose) are characterised by low-fold differences. The latter is exemplified by the low but significant differences in transcription of genes in the pentose phosphate pathway itself (Table 2). Large differences are also found when comparing transcript levels of genes encoding extracellular enzymes, expression of which are specifically induced by particular carbon sources (Table 3). Whereas the basal process of protein secretion is governed by a multitude of proteins and protein complexes, many of which are conditionally expressed, but only with small differences in transcript level (Table 4). A solid context of identical specific growth rates has allowed us to study such small, but significant, differences in gene expression between two similar growth conditions. Andersen *et al*. [4] defined, for three *Aspergillus *species (incl. *A. niger*), a conserved transcriptional response to xylose as carbon source compared to glucose (degradation product of maltose). This list does not include pentose phosphate pathway (PPP) genes, showing that the substrate-effect on expression of these genes may be specific for *A. niger *or simply that it is difficult to observe such effects without control of the specific growth rate.

**Table 2:** Xylose utilisation metabolic genes with significantly higher expression on xylose compared to maltose.

ORF	gene name	encoded enzyme (homolog)	fold difference	signal xylose	SD	Signal maltose	SD	p	FDR
Xylose conversion:									
An01g03740	*xyrA*	D-xylose reductase	15.9	9,267	511	1,048	1,033	4.1·10^-3^	4.5·10^-2^
An07g03140		xylulokinase (Xks1 – *S. cerevisiae*)	16.0	916	103	58	34	2.3·10^-5^	1.3·10^-3^
									
Pentose phosphate pathway – nonoxidative phase:									
An08g06570		transketolase (Tkl1 – *S. cerevisiae*)	1.4	5,410	439	3,522	448	1.6·10^-4^	4.7·10^-3^
An07g03850		transaldolase (Tal1 – *S. cerevisiae*)	1.4	5,127	615	3,257	566	1.6·10^-4^	4.7·10^-3^
An07g03160		transaldolase (TalB – *Synechocystis *sp.)	8.9	551	32	60	26	2.1·10^-5^	1.2·10^-3^
									
Glycolysis/gluconeogenesis:									
An16g05420		glucose-6-phosphate isomerase (Pgi1 – *S. cerevisiae*)	1.7	1,481	278	789	61	2.5·10^-3^	3.2·10^-2^
									
Pentose phosphate pathway – oxidative phase:									
An02g12140	*gsdA*	glucose-6-phosphate dehydrogenase	1.5	1,588	159	966	111	4.5·10^-5^	2.0·10^-3^

ORF = identifier for open reading frame in *A. niger *CBS513.88 genome sequence [3]; gene name in *A. niger*; enzyme encoded by ORF (gene product and species name in parenthesis indicate closest ORF-homolog with characterized function); fold difference reflects ratio of normalized transcript levels on xylose compared to maltose (xylose/maltose); mean signal values of six experiments on each carbon source and standard deviations (SD) is given in Affymetix units; significance of each observation is given by p-value (p) and the Benjamini-Hochberg false discovery rate (FDR).

**Table 3:** The 10, most highly and differentially expressed, secreted-carbohydrase genes on xylose or maltose.

ORF	gene name	encoded enzyme (homolog)	fold difference	signal xylose	SD	signal maltose	SD	p	FDR
High expression on maltose (maltose/xylose):									
An03g06550	*glaA*	glucoamylase	3.5	4,175	574	*12,892	805	1.8·10^-8^	4.5·10^-5^
An04g06920	*agdA*	extracellular alpha-glucosidase	25.0	468	54	10,460	1,117	6.8·10^-12^	8.5·10^-8^
An11g03340	*aamA*	acid alpha-amylase	100.5	37	11	3,202	356	3.6·10^-10^	1.1·10^-6^
An09g00260	*aglC*	alpha-galactosidase	4.9	450	61	1,973	310	1.8·10^-8^	1.4·10^-5^
An12g08280	*inu1*	extracellular exo-inulinase	25.2	57	9	1,279	44	9.9·10^-10^	2.4·10^-6^
									
High expression on xylose (xylose/maltose):									
An01g00780	*xynB*	endo-1,4-xylanase	22.3	7,846	1,169	422	346	1.5·10^-4^	4.6·10^-3^
An01g09960	*xlnD*	xylosidase	43.3	5,260	205	182	185	1.7·10^-4^	5.1·10^-3^
An14g02760	*eglA*	endo-glucanase	81.3	4,079	476	56	47	4.0·10^-7^	1.1·10^-4^
An14g05800	*aguA*	alpha-glucuronidase	45.0	3,619	433	81	45	1.4·10^-6^	2.2·10^-4^
An09g03300	*axlA*	alpha-xylosidase	10.3	3,365	529	438	398	2.5·10^-3^	3.2·10^-2^

ORF = identifier for open reading frame in *A. niger *CBS513.88 genome sequence [3]; gene name in *A. niger*; enzyme encoded by ORF (gene product and species name in parenthesis indicate closest ORF-homolog with characterized function); fold difference reflects ratio of normalized transcript levels (maltose/xylose or xylose/maltose); mean signal values of six experiments on each carbon source and standard deviations (SD) is given in Affymetix units; significance of each observation is given by p-value (p) and the Benjamini-Hochberg false discovery rate (FDR).

*signal of An03g06550-probeset appears saturated, leading to underestimation of *glaA*-transcription in maltose-limited chemostat cultures.

**Table 4:** Differential expression of secretory pathway genes.

ORF	gene name	homologous protein in *S. cerevisiae*	fold difference **maltose/xylose**	p	FDR
Translocation to ER:					
**An07g05800**		**SRP14 (YDL092w) – signal recognition particle SU**	1.3	1.7·10^-4^	4.9·10^-3^
An15g06470*		**signal sequence receptor, αSU (*Botryotinia fuckeliana*)**	1.7	1.6·10^-5^	1.1·10^-3^
**An03g04340**		**SEC61 (YLR378c) – SEC61 complex SU**	1.7	2.1·10^-5^	1.2·10^-3^
An01g11630*		SSS1 (YDR086c) – SEC61 complex SU	1.5	1.1·10^-3^	1.8·10^-2^
An01g13070*		**SEC63 (YOR254c) – SEC63 complex SU**	1.6	6.9·10^-5^	2.7·10^-3^
An02g01510		SEC62 (YPL094c) – SEC63 complex SU	1.7	1.5·10^-3^	2.3·10^-2^
An16g08830*		SEC66 (YBR171w) – SEC63 complex SU	1.8	3.5·10^-4^	8.3·10^-3^
					
Cleavage of signal sequence:					
**An16g07390**		**SPC2 (YML055w) – signal peptidase complex SU**	1.7	2.5·10^-5^	1.4·10^-3^
An09g05420**		**SPC3 (YLR066w) – signal peptidase complex SU**	1.7	2.1·10^-5^	1.2·10^-3^
An01g00560*		SEC11 (YIR022w) – signal peptidase complex SU	1.7	1.7·10^-3^	2.5·10^-2^
					
Glycosylation:					
**An16g04330**		**DPM1 (YPR183w) – dolichol phosphate mannose synthase**	1.6	2.3·10^-5^	1.3·10^-3^
An14g00270		dolichyl-phosphate mannosyltransferase (*B. fuckeliana*)	1.4	7.5·10^-4^	1.4·10^-2^
An03g04410**		ALG5 (YPL227c) – dolichyl-phosphate glucosyltransferase	1.6	2.8·10^-4^	7.2·10^-3^
An02g03240*		ALG7 (YBR243c) – *N*-acetyl-glucosaminephosphotransferase	1.7	2.2·10^-4^	6.0·10^-3^
An14g05910*		ALG2 (YGL065c) – mannosyltransferase	1.9	1.3·10^-3^	2.1·10^-2^
An04g03130		mannose-phosphate-dolichol utilization protein (*Mus musculus*)	1.6	2.1·10^-4^	5.7·10^-3^
An08g07020		ALG9 (YNL219c) – mannosyltransferase	1.4	4.2·10^-3^	4.6·10^-2^
An02g12630		ALG6 (YOR002w) – glucosyltransferase	1.3	1.7·10^-3^	2.5·10^-2^
An02g14940*		**RFT1 (YBL020w) – flippase**	1.5	7.6·10^-5^	2.8·10^-3^
An02g14560*		**OST1 (YJL002c) – oligosaccharyltransferase complex, αSU**	1.7	1.7·10^-4^	5.0·10^-3^
An07g04190*		**WBP1 (YEL002c) – oligosaccharyltransferase complex, βSU**	1.7	1.2·10^-4^	3.8·10^-3^
An18g03920*		**OST2 (YOR103c) – oligosaccharyltransferase complex, εSU**	1.5	1.4·10^-4^	4.4·10^-3^
**An02g14930**		**OST3 (YOR085w) – oligosaccharyltransferase complex, γSU**	1.5	9.7·10^-5^	3.4·10^-3^
**An16g08570**		**STT3 (YGL022w) – oligosaccharyltransferase complex, SU**	1.7	4.1·10^-5^	1.9·10^-3^
An04g06990		MNS1 (YJR131w) – class I α-mannosidase	1.3	2.0·10^-3^	2.8·10^-2^
An06g01510		class I α-mannosidase (*Aspergillus fumigatus*)	1.7	2.1·10^-4^	5.7·10^-3^
An12g00340		ER glucosyl hydrolase, Edem (*A. fumigatus*)	1.4	1.0·10^-3^	1.7·10^-2^
**An07g04940**		**HOC1 (YJR075w) – α-1,6-mannosyltransferase**	1.5	1.9·10^-5^	1.2·10^-3^
**An16g08490**		**PMT4 (YJR143c) – *O*-mannosyltransferase**	1.3	1.5·10^-4^	4.7·10^-3^
An15g04810		MNT2 (YGL257c) – α-1,3-mannosyltransferase	0.7	1.4·10^-3^	2.2·10^-2^
An08g04450		GDA1 (YEL024w) – guanosine diphosphatase	1.3	7.7·10^-4^	1.4·10^-2^
					
Folding:					
An02g14800**	***pdiA***	**PDI1 (YCL043c) – protein disulfide isomerase**	1.8	1.1·10^-5^	3.6·10^-3^
An02g05890	*epsA*	thioredoxin domain protein, TXNDC5 (*Homo sapiens*)	1.3	1.3·10^-3^	2.1·10^-2^
An18g02020*	*tigA*	protein disulfide isomerase	1.6	2.3·10^-4^	6.1·10^-3^
An01g04600**	*prpA*	MPD1 (YOR288c) – protein disulfide isomerase	1.9	6.0·10^-4^	1.2·10^-2^
An16g07620**		ERO1 (YML130c) – thiol oxidase	1.5	3.1·10^-3^	2.1·10^-2^
An18g04260**		**HUT1 (YPL244c) – UDP-galactose transporter**	1.6	1.5·10^-4^	4.6·10^-3^
An08g07810		FAD1 (YDL045c) – FAD synthase	1.3	1.3·10^-3^	2.1·10^-2^
An11g04180**	***bipA***	**KAR2 (YJL034w) – ER chaperone**	2.2	5.0·10^-5^	2.2·10^-3^
An18g06470		ERJ5 (YFR041c) – ER located DNA-J protein	1.5	3.4·10^-4^	8.2·10^-3^
An01g13220**		LHS1 (YKL073w) – ER lumen Hsp70 chaperone	1.9	2.8·10^-4^	7.1·10^-3^
An01g06670		FPR2 (YDR519w) – peptidyl-prolyl isomerase	1.7	1.9·10^-3^	2.7·10^-2^
					
Trimming and quality control of N-glycosylated folded proteins:					
An15g01420*		**CWH41 (YGL027c) – alpha glucosidase I**	1.7	3.3·10^-6^	3.8·10^-4^
**An09g05880**		**ROT2 (YBR229c) – glucosidase II, αSU**	1.5	1.1·10^-5^	8.5·10^-4^
An13g00620*		**GTB1 (YDR221w) – glucosidase II, βSU**	1.7	2.6·10^-5^	1.4·10^-3^
An01g08420**	***clxA***	**CNE1 (YAL058w) – calnexin**	2.3	6.5·10^-6^	5.8·10^-4^
					
Vesicular transport of proteins between ER and Golgi:					
**An04g00360**		**SEC13 (YLR208w) – COPII complex SU**	1.4	2.2·10^-5^	1.3·10^-3^
An02g01690		SEC31 (YDL195w) – COPII complex SU	1.6	2.7·10^-4^	7.0·10^-3^
An08g10650		SEC24 (YIL109c) – COPII complex SU	1.5	1.6·10^-3^	2.3·10^-2^
**An01g04730**		**SEC23 (YPR181c) – COPII complex SU**	1.6	1.4·10^-4^	4.3·10^-3^
An08g03590		EMP24 (YGL200c) – COPII vesicle membrane component	1.4	2.8·10^-3^	3.4·10^-2^
An04g01780		ERP1 (YAR002c-a) – COPII vesicle component	1.5	1.3·10^-3^	2.1·10^-2^
**An09g05490**		**ERP3 (YDL018c) – p24 family protein**	1.5	1.3·10^-4^	4.1·10^-3^
**An04g08830**		**EMP47 (YFL048c) – COPII vesicle membrane component**	1.4	1.2·10^-5^	8.9·10^-4^
**An08g03960**		**ERV29 (YGR284c) – glycoprotein cargo receptor**	1.5	2.1·10^-5^	1.2·10^-3^
An02g04250*		**vesicular integral-membrane protein (*Pyrenophora tritici-repentis*)**	1.6	7.6·10^-5^	2.8·10^-3^
An07g09960		BET1 (YIL004c) – v-SNARE	1.3	5.0·10^-3^	5.1·10^-2^
An07g02170		BOS1 (YLR078c) – v-SNARE	1.4	1.8·10^-3^	2.6·10^-2^
An08g06780*		USO1 (YDL058w) – SNARE complex assembly protein	1.8	2.2·10^-4^	6.0·10^-3^
An02g06870		RAD50-interacting protein 1 (*M. musculus*)	1.2	2.3·10^-3^	3.0·10^-2^
An04g01990		centromere protein ZW10 (*Gallus gallus*)	1.3	1.1·10^-3^	1.8·10^-2^
An04g08690		GSG1 (YDR108w) – TRAPP complex SU	1.3	4.9·10^-3^	5.0·10^-2^
**An04g06090**		**BET4 (YJL031c) – geranylgeranyltransferase, α SU**	1.5	8.1·10^-6^	6.8·10^-4^
An03g04940**		**ERV41 (YML067c) – involved in COPII vesicle fusion**	2.1	1.2·10^-5^	9.0·10^-4^
An01g04320*		**ERV46 (YAL042w) – involved in COPII vesicle fusion**	1.8	1.7·10^-5^	1.1·10^-3^
**An08g00290**		**RUD3 (YOR216c) – Golgi-matrix protein**	1.4	2.8·10^-5^	1.5·10^-3^
**An08g03690**		**ARF2 (YDL137w) – ADP-ribosylation factor**	1.3	1.8·10^-5^	1.1·10^-3^
An07g02190		SEC7 (YDR170c) – guanine nucleotide exchange factor	1.3	2.7·10^-3^	3.4·10^-2^
An18g02490		GEA2 (YEL022w) – guanine nucleotide exchange factor on ARF	1.3	6.8·10^-4^	1.3·10^-2^
**An16g02460**		**COP1 (YDL145c) – COPI complex, α SU**	1.4	3.5·10^-5^	1.7·10^-3^
**An07g06030**		**SEC21 (YNL287w) – COPI complex, γ SU**	1.4	2.9·10^-5^	1.5·10^-3^
**An08g06330**		**SEC28 (YIL076w) – COPI complex, ε SU**	1.5	3.2·10^-5^	1.6·10^-3^
**An08g03270**		**SEC26 (YDR238c) – COPI complex, β SU**	1.4	1.8·10^-4^	5.3·10^-3^
An02g05870		SEC27 (YGL137w) – COPI complex, β ' SU	1.5	5.4·10^-4^	1.1·10^-2^
**An02g02830**		**RER1 (YCL001w) – retention of ER membrane proteins**	1.3	1.1·10^-4^	3.8·10^-3^
An04g05250*		RER2 (YBR002c) – retention of ER membrane proteins	1.2	3.7·10^-3^	4.2·10^-2^
**An07g07340**		**ERD2 (YBL040c) – ER retention HDEL-receptor**	1.4	1.1·10^-4^	3.8·10^-3^
					
Other processes in the secretory pathway:					
An11g02650		AGE2 (YIL044c) – ARF GTPase activating protein effector	1.1	3.6·10^-3^	4.0·10^-2^
An16g03590		SEC14 (YMR079w) – phosphatidylinositol/-choline transfer protein	1.2	3.3·10^-3^	3.8·10^-2^
An04g02070		CHC1 (YGL206c) – clathrin, heavy chain	1.2	2.7·10^-3^	3.4·10^-2^
An16g02490		APL2 (YKL135c) – β-adaptin	1.5	3.3·10^-3^	3.8·10^-2^
An16g03010		VPS4 (YPR173c) – vacuolar protein sorting AAA-ATPase	0.8	3.1·10^-3^	3.7·10^-2^
An02g05380		VPS33 (YLR396c) – vacuolar protein sorting	1.3	2.5·10^-4^	6.5·10^-3^
An14g05130		VPS16 (YPL045w) – vacuolar protein sorting	0.8	4.0·10^-3^	4.4·10^-2^
An01g02910		VPS52 (YDR484w) – vacuolar protein sorting	1.4	4.2·10^-3^	4.6·10^-2^
An02g11720		AMS1 (YGL156w) – vacuolar α-mannosidase	0.8	2.3·10^-3^	3.0·10^-2^
An06g01200		EMP70 (YLR083c) – conserved endosomal membrane protein	1.3	3.2·10^-4^	7.8·10^-3^
An03g06900		SEC10 (YLR166c) – exocyst complex SU	1.2	3.5·10^-4^	8.3·10^-3^
An02g04030		EXO70 (YJL085w) – exocyst complex SU	1.3	3.7·10^-3^	4.1·10^-2^
An01g11960		BFR1 (YOR198c) – component of mRNP complex	1.4	2.3·10^-4^	6.1·10^-3^
An04g01950		STE24 (YJR117w) – zinc metalloprotease	1.3	2.7·10^-4^	6.9·10^-3^
An07g10050		microtubule binding protein HOOK3 (*A. fumigatus*)	1.2	2.7·10^-3^	3.4·10^-2^
					
Protein misfolding (UPR and ER associated degradation):					
**An08g01480**		**TRL1 (YJL087c) – tRNA ligase**	0.7	1.1·10^-4^	3.7·10^-3^
An01g07900	*cpcA*	GCN4 (YEL009c) – bZIP transcription factor	0.8	2.6·10^-3^	3.3·10^-2^
An11g11250*		**protein kinase inhibitor p58 (*Rattus norvegicus*)**	1.6	4.7·10^-5^	2.1·10^-3^
An01g08980		ORM1 (YGR038w) – conserved ER protein	0.7	3.8·10^-4^	8.8·10^-3^
An15g00640		DER1 (YBR201w) – involved in ER associated protein degradation	1.4	2.2·10^-3^	3.0·10^-2^
An16g07970		HRD1 (YOL013c) – ubiquitin-protein ligase	1.3	8.3·10^-4^	1.5·10^-2^
**An01g12720**		**HRD3 (YLR207w) – ubiquitin-protein ligase**	1.6	6.4·10^-5^	2.5·10^-3^
An09g06110		UBC7 (YMR022w) – ubiquitin conjugating enzyme	1.2	3.0·10^-3^	3.6·10^-2^
An04g01720		HLJ1 (YMR161w) – DnaJ co-chaperone	1.3	2.6·10^-4^	6.8·10^-3^

ORF = identifier for open reading frame in *A. niger *CBS513.88 genome sequence [3]; gene name in *A. niger*; protein encoded by ORF-homolog in *S. cerevisiae *and yeast protein function if available; fold difference reflects ratio of normalized transcript levels on maltose compared to xylose (maltose/xylose); significance of each observation is given by p-value (p) and the Benjamini-Hochberg false discovery rate (FDR).

Bold indicates observations with very high significance (FDR ≤ 0.005).

* and ** denote genes with increased transcription during ER-stress with 2/3 or 3/3 types of protein folding stress [15], repectively.

**Figure 4:**
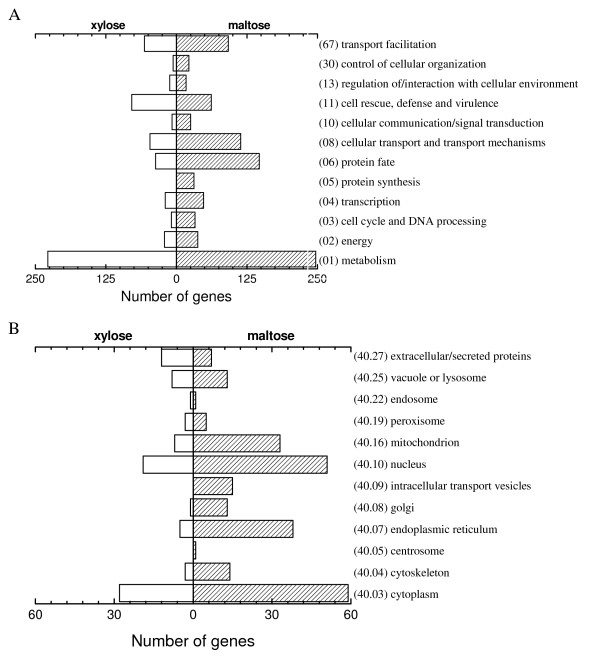
**Functional classification of differentially expressed genes (open bars indicate number of genes with higher transcript levels on xylose; hatched bars represent genes higher expressed on maltose)**. (A) Representation of major functional categories (Funcat) among differentially expressed genes. (B) Subcellular localization of differentially expressed genes. Unclassified ORFs: high on xylose, 40% (213/528); high on maltose, 30% (214/712).

Using the FunCat annotation tool [3,17], an overview of up- or down-regulation of major functional classes during maltose-limitation (compared to xylose-limitation) is given in Fig. 4A. "Metabolism (01)" and "Transport facilitation (67)" were represented as two major functional classes with differentially expressed genes, reflecting need for uptake and metabolism of two different carbon sources. The efficient energy-metabolism on xylose, evident from the high biomass yield and high specific oxygen consumption rate of xylose-limited cultures (Table 1), may be rooted in enhanced expression of genes encoding glucose-6-phosphate dehydrogenase, xylose conversion enzymes and the nonoxidative phase of pentose phosphate pathway (Table 2).

In the carbon metabolism category were also genes encoding secreted carbohydrases (Table 3). Highly expressed genes on maltose include genes encoding the acid amylase (AamA), glucoamylase (GlaA) and the alpha-glucosidase (AgdA), enzymes which have also been identified as highly induced genes, when grown on maltose in batch cultures [18]. On maltose, *glaA *displayed the highest transcript level of all genes. Array signals and Northern blot analysis indicated that probes for *glaA *were saturated, resulting in an underestimation of fold difference [see Additional file 4]. The list of highly-induced genes on xylose, contains enzymes involved in xylan degradation. Induction of *xlnD*, *eglA *and *aguA *by xylose in a XlnR-dependent way have been described [19]. High expression of *axlA *on xylose supports its proposed function as an alpha-xylosidase [18]. Strong induction of *xynB *on xylose has not been reported before, but its function as an endo-xylanase fits well with its expression profile.

Lipid and sterol biosynthesis and fatty acid metabolic genes also constituted a large group in differentially expressed metabolic genes [Additional file 5]. Perhaps most striking in this group, was the 12-fold increase in transcript level on maltose of a highly expressed and apparently secreted lipase (An16g01880; FDR = 2.9·10^-4^). In general, several genes involved in biosynthesis of ergosterol and phospholipids were upregulated on maltose, while inositol and choline biosynthesis genes were downregulated. Among these genes, we find a homolog of *ino1 *(An10g00530), encoding inositol-1-phosphate synthase, a key enzyme in inositol biosynthesis in *S. cerevisiae*. The differential expression of genes in this category indicate changes (proliferative and/or compositional) in membrane components or energy storage. Intermediates of phospholipid and inositol metabolic pathways also play important roles in cell signalling and global regulatory pathways [20].

**Figure 5:**
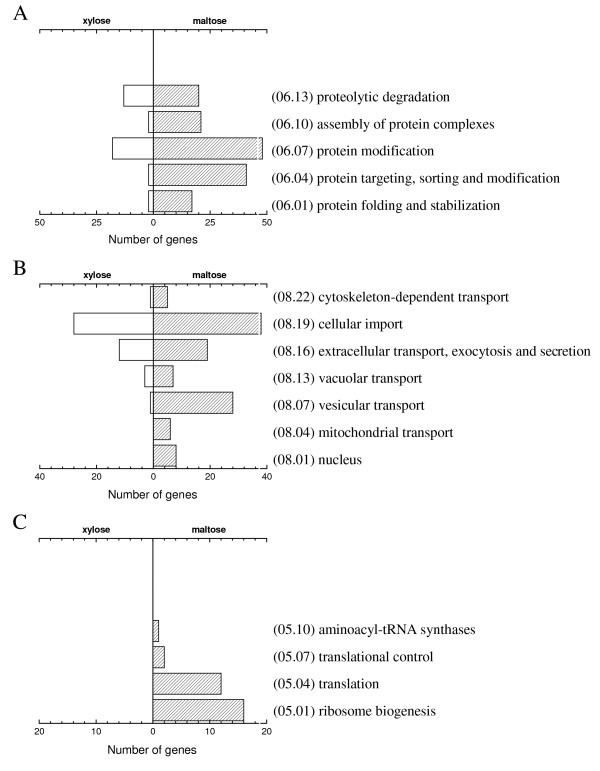
**Differentially expressed genes in sub-Funcats of Fig. **4A. (A) 06 – Protein fate. (B) 08 – Cellular transport and transport mechanisms. (C) 05 – Protein synthesis.

"Protein fate (06)" and "Cellular transport and transport mechanisms (08)" comprise most genes of the secretory pathway, and were the second and third largest functional categories of genes with higher transcript level on maltose than on xylose (Fig. 4A). "Subcellular localization (40)" also points to maltose-induced upregulation of the secretory pathway, since many genes are associated with organelle compartments, like the ER, Golgi, transport vesicles, nuclei and mitochondria (Fig. 4B). Subcategories in Fig. 5(A, B) clearly illustrate the uniform upregulation of genes in secretory processes on maltose compared to xylose. Genes in "Protein synthesis (05)" were only upregulated on maltose. Among these are several genes involved in ribosome biogenesis, translation initiation and polypeptide elongation (Fig. 5C and [Additional file 6]). Thus, the FunCat overview reveals that upregulation of genes involved in protein synthesis and secretion on maltose is a major difference between the two substrates.

### Maltose induces expression of secretory pathway genes

Maltose induces transcription of at least 90 defined secretory pathway genes (Table 4), consistent with higher productivity of extracellular protein and very high expression-level of glucoamylase observed in maltose-limited chemostat cultures (Table 1 and 3). We could only identify a few genes (six) in the secretory pathway, which were higher expressed on xylose (Table 4). Among the genes with most significant (FDR ≤ 0.005) higher transcript level on maltose are those encoding essential subunits of the Sec61/-63 translocation complex; subunits of the signal peptidase complex; *N*-glycosylation enzymes like dolichol-phosphate-mannosyl flippase and most subunits of the oligosaccharyltransferase (OST) complex; important chaperones and foldases (*bipA*, *pdiA *and *clxA*) with well characterized functions in *A. niger *[10,21,22]; calnexin (*clxA*) is involved in folding and quality control of *N*-glycosylated proteins together with glucosidase I and II, which also display some of the most significant upregulation. The transcriptomic profiles support the recent phylogenetic prediction [18] that An09g05580 encodes the alpha subunit of glucosidase II; since the significance, fold difference (maltose/xylose) and expression level of this ORF is almost identical to the known beta-subunit, while these variables are quite different in the five other candidate ORFs given by Pel *et al*. [3]. ER-associated degradation (ERAD) is represented among genes with maltose-induced transcription. ER to Golgi vesicular transport of glycosylated proteins is upregulated on maltose, shown by higher transcript levels of genes encoding key COPII coat proteins and lectin cargo receptor proteins.

Genes involved in retrograde vesicular transport (COPI coatamers) and recycling of ER proteins are also higher expressed, and together with upregulation of ER associated degradation (ERAD) of glycoproteins genes, this suggest a general increase in capacity of at least the ER related processes in the secretory pathway. The functions, upregulated during higher protein secretion on maltose, are thus mainly localised in the proximal part of the secretory pathway. Whereas, there was little differential expression of genes encoding components in the more distal parts of the secretory pathway, i.e. trans-Golgi, late secretory vesicles and exocytosis, and endocytosis (Table 4).

It has been suggested, that efficient protein secretion in filamentous fungi may be obtained through existence of alternative secretory pathways [23]. Our study, on the other hand, suggests that *A. niger *can adapt the secretory capacity by transcriptional regulation of central processes in synthesis, folding and transport of glycoproteins to permit efficient secretion of extracellular proteins, like e.g. glucoamylase (even at wild type levels of secretion). This conclusion is consistent with the observation that the activities of three *N*-glycosylation enzymes(*N*-acetylglucosaminyl phosphate transferase, dolichol phosphoglucosyl synthase and dolichol phosphomannosyl synthase) are 2–3 folds higher at growth on maltose-containing medium (maltodextrin) of a glucoamylase hyper-producing *A. niger *strain compared to a wild type strain [14]. Furthermore, the hyper-producing strain displays wild type activity (low) of *N*-acetylglucosaminyl phosphate transferase, when both strains are cultivated on xylose [14], which is non-inducing for *glaA *expression. Some notable analogies to maltose-induction of secretory pathway genes and *glaA *expression in *A. niger *were observed by Saloheimo *et al*. [24] in the filamentous fungus *Trichoderma reesei*. They showed that expression of *pdi-1 *is induced by cellulose and that this correlates with increased expression of cellulase genes and secretion of their gene-products. In mammals it has been observed that glucose induces expression of several secretory pathway genes in pancreatic β-cells [25]. Furthermore, differentiation into a dedicated secretory cell (e.g. a plasma cell), involves concerted transcriptional upregulation of the secretory pathway via the unfolded protein response (UPR). This phenomenon has been termed a physiological UPR. It is mediated by the master regulator of secretion, Xbp1, which is an ortholog of the Hac1 UPR-regulator in fungi [26,27]. From the functional classification and subcellular localization of differentially expressed genes, it is concievable that similar changes take place in the maltose-limited cultures of *A. niger*.

### Comparison with ER-stress and UPR in fungi

In *S. cerevisiae*, the UPR effect on gene transcription has been described for induction with the chemical ER-stressors, DTT and tunicamycin [28], and forced expression of the active form of the UPR transcription factor, Hac1p [29]. A common response to ER-stress in yeast and growth on maltose (this study), is transcriptional upregulation of genes involved in ER-associated processes, such as translocation, *N*-glycosylation, ERAD and anterograde vesicle transport. The present study even adds to the list of ER-associated genes which are upregulated in response to increased load on the secretory pathway (Table 4). Kitama *et al*. [29] reported that the UPR led to down-regulation of 15 genes. Of these, a ferrous iron transporter homolog (An01g08960) was significantly lower expressed on maltose (fold difference maltose/xylose = 0.07, FDR = 3.7·10^-4^).

UPR in *S. cerevisiae *does not seem to induce transcription of genes involved in trimming and quality control of glycoproteins or in retrograde vesicular transport of proteins from Golgi to ER [28,29]. Travers *et al*. [28] suggested, that this observation indicates that the UPR in *S. cerevisiae *functions to relieve stress – not to mediate a general increase in secretion capacity. This is in contrast to the present study, where calnexin, subunits of glucosidase I and II and several genes involved in retrograde transport were consistently upregulated during growth on maltose (Table 4). A common theme of the UPR is also changes in transcription of lipid and inositol metabolism. Although growth on maltose led to many significant changes in transcription of both lipid and inositol pathway genes, the changes did not resemble those induced by UPR in *S. cerevisiae *[28,29]. Transcription of *ino1 *was upregulated during constitutive Hac1p-induced UPR [29], while the homolog (An10g00530) of this gene was down-regulated in *A. niger *growing on maltose. However, similar observations of *ino1 *transcription have been made in *S. cerevisiae *strains with secretory pathway defects, which induce the UPR [30].

A recent extensive study of the UPR in *A. niger *[15] defined common transcriptional responses to treatment with the chemicals, tunicamycin or DTT, and forced secretion of a heterologous protein; conditions, which lead to accumulation of unfolded proteins and ER-stress. The three types of ER-stress induced expression of many genes encoding major functions in the secretory pathway. The 11 genes induced by all three ER-stress conditions [15] were all higher expressed on maltose in this study. In fact, of the genes listed in Table 4, 29 are represented in the list of 34 secretory pathway genes induced by two or all three of the above mentioned stress conditions. Comparing the functions encoded by the ER-stress-induced genes to those with increased expression on maltose, we find that most of the 29 genes are involved in processes early in the protein secretion pathway. These functions are similar to the yeast UPR response and encompass subunits of the translocation complex, signal peptidase, *N*-glycosylation proteins, foldases and chaperones. In addition to the yeast profiles, calnexin and glucosidase I and II subunits were also upregulated by ER-stress in *A. niger*. Interestingly, a homolog (An11g11250) of the mammalian interferon induced protein kinase, p58^IPK^, was significant higher expressed both on maltose (Table 4) and in two of the three previously described ER-stress conditions [15]. In mammals, Xbp1 enhances expression of p58^IPK^. This has been suggested as a mechanism to antagonise PERK-mediated repression of global protein synthesis during physiological UPR in secretory cells [26]. *A. niger *lacks an obvious homolog of PERK. It consequently seems probable, that the target of the p58^IPK^-like protein is a homolog of another mammalian protein kinase, like PKR (in *Homo sapiens*) [31]. The protein encoded by the *A. niger *PKR-homolog (An17g00860) has high identity (31% over 1682 residues) to the protein kinase, Gcn2p, in *S. cerevisiae*. It has been shown in other ascomycete fungi, that Gcn2p homologs have roles in regulation of global protein synthesis and cell cycle progression [32,33]. It is tempting to speculate that p58^IPK ^in *A. niger *can exert derepression of protein synthesis through a homolog of Gcn2p. In *S. cerevisiae*, Gcn2p plays additional role as a positive regulator of translation of the amino acid starvation transcription factor Gcn4p, which apparently regulates transcription of many UPR induced genes [34]. In our study, CpcA (An01g07900), a homolog of Gcn4p, was down-regulated on maltose, much in contradiction with its putative function as positive regulator of transcription of several UPR target genes in the secretory pathway. The differential expression of key elements of the UPR, such as homologs of p58^IPK ^and Gcn4p (CpcA), suggests that upregulation of the secretory pathway on maltose occurs via Gcn4p-independent mechanisms.

There were clear differences between the UPR induced by artificial ER-stressors [15], and the physiological response to maltose. For example, during artificially induced ER-stress, very few genes, encoding ERAD, COPII and especially COPI vesicular transport proteins, were commonly upregulated. Guillemette *et al*. [15] also observed changes in expression of fatty acid and lipid metabolism genes. One gene (An02g13420) with similarity to an acetyl-coenzyme A transporter was consistently upregulated in all three stress conditions and during growth on maltose; again indicating relation between lipid metabolism and the secretory pathway. This similarity is in contrast to the remaining of the differentially expressed genes in this category; of the seven genes down-regulated by ER-stress we observed four upregulated on maltose. This included the on maltose highly-expressed, secreted or organelle-associated, lipase (An16g01880). This gene was upregulated during ER-stress caused by expression of a heterologous protein, but not in presence of DTT or tunicamycin [15]. A homolog of *erg24*, a gene involved in ergosterol biosynthesis in *S. cerevisiae*, was opposite many other putative ergosterol biosynthesis genes down-regulated on maltose, and its transcription was also lower during expression of the heterologous protein [15]. This shows that although the ER-stress responses share a core of similarly expressed functions, expression of other functions are specific to the nature of the ER-stress.

### UPR – part of a differentiation programme in development of high secretion capacity?

Despite many similarities to the fungal ER-stress responses defined by treatment with harsh chemicals or constitutive active expression of the UPR transcription factor, Hac1, or of heterologous proteins, the physiological state of the fungal cells in our study was very different as maltose induced overall protein secretion and transcription of highly expressed hydrolases. Notably, differences in expression of genes in lipid metabolism and in protein synthesis show clear differences between maltose-induced transcription of secretory pathway genes and the chemical-induced UPR. This indicates that what looks like a response to secretion stress, may reflect a more general mechanism not only to alleviate accumulation of unfolded proteins in more extreme conditions, but also to modulate secretion capacity in response to the conditional need for extracellular enzymes. The higher expression level of secretory pathway genes on maltose resembles physiological UPR as described in mammalian cells with high secretion activity a feature which could partly explain why filamentous fungi are efficient enzyme secretors. However, the HacA transcription factor is an integral part of the UPR, and simple evaluation of splicing of HacA mRNA, using RT-PCR [Additional file 7], did not reveal a difference between growth on maltose or xylose nor was there any difference in transcription of *hacA*. This indicates that the carbon source dependent differential expression of secretory pathway genes involves additional factors than HacA.

This investigation not only presents supporting evidence for a concerted transcriptional regulation of many secretory pathway genes, but also a challenging task with respect to identification of key regulators. Regulatory elements of basal cellular processes may be involved, as many genes involved in RNA-processing and basal regulation of transcription from RNA polymerase II were differentially expressed [Additional file 8]. One very intriguing gene (An07g03760) was upregulated on maltose with same level of significance and fold difference as the most significant upregulated secretory pathway genes. It encodes a highly conserved homolog of the P100 EBNA-2 transcriptional co-activator, which is abundantly expressed in endo-and exocrine tissues in mammals [35,36]. The homolog of this gene was also higher expressed in *T. reesei *during secretion stress [37], indicating that this transcriptonal profile is conserved among filamentous fungi. Despite a high degree of conservation in all eukaryotic lineages no homolog is found in the yeast *S. cerevisiae*. Elements of some cellular functions, like protein secretion, may be more conserved between higher eukaryotes and filamentous fungi, compared to the in many ways reduced ascomycete, *S. cerevisiae*. Consequently, a better understanding of processes and regulation of protein secretion in filamentous fungi may require comparison to secretory cells of higher eukaryotes.

## Conclusion

We provide evidence for carbon source induced, growth rate independent and concerted transcriptional regulation of genes encoding proteins in the secretory pathway in *A. niger*. We hypothesize that the fungal cell has a principal mechanism for co-ordinated regulation of basal anabolic pathways to modulate capacity of the secretory pathway. The main perspectives of our results are identification of putative regulatory elements, which coordinate expression of genes involved in protein synthesis and secretion, and increased understanding of transcriptional regulation of the secretory pathway in more normal physiological conditions. We show that an appropriate experimental approach allows study of subtle changes in expression of genes involved in important cellular processes. Furthermore, studies of lower eukaryotes, like *A. niger*, can be used to study functions and processes which are conserved in higher eukaryotes, with advantages in ease of propagation and ability to control growth and environmental variables.

## Methods

### Strains and inoculum

*Aspergillus niger *AB94-85 [38] and ABGT1026 [39] were cultivated in triplicate xylose- and maltose-limited chemostat cultures. Both strains are derived from N400 (= NRRL3, ATCC9029, CBS120.49).

Conidia for inoculation of bioreactor cultures were harvested from solidified Complete Medium (CM), which contained (per liter): 6.0 g NaNO_3_, 1.5 g KH_2_PO_4_, 0.5 g KCl, 0.5 g Mg SO_4_·7H_2_O, 1.0 g casamino acids, 5.0 g yeast extract, 20 g agar and 1 ml trace metal solution. The trace metal solution contained per liter, 10 g EDTA, 4.4 g ZnSO_4_·7H_2_O, 1.01 g MnCl_2_·4H_2_O, 0.32 g CoCl_2_·6H_2_O, 0.315 g CuSO_4_·5H_2_O, 0.22 g (NH_4_)_6_Mo_7_O_24_·4H_2_O, 1.47 g CaCl_2_·2H_2_O and 1 g FeSO_4_·7H_2_O (modified from composition given by Vishniac and Santer [40]). pH of CM was adjusted to 5.8 with NaOH and autoclaved. The major carbon source, xylose, was autoclaved separately and added to give a final concentration of 1% (w/v). Conidia from a -80°C stock culture were spread over moist sterile filter paper discs, which were placed on CM plates. Filter paper discs facilitate spore harvest and increase yield of conidia. The solid medium cultures were incubated for three days at 30°C and then for five to seven days at 25°C to enhance conidiation. Spore plates were stored for no more than six months at 4°C. Conidia were harvested from filter paper discs with a sterile detergent solution containing 0.05% (w/v) Tween 80 and 0.9% (w/v) NaCl.

### Continuous cultivation

Carbon-limited chemostat cultures were performed in a Variomixing Bioreactor [41]. This bioreactor was designed especially for cultivation of filamentous microorganisms, which have a tendency to adhere to and grow on surfaces. The Variomixing Bioreactor possesses a number of features, which reduce biomass adhesion and wall growth, and thus maintains high culture homogeneity during prolonged cultivation of filamentous fungi. A more detailed description of the Variomixing Bioreactor and associated equipment used for continuous cultivation is available elsewhere [41,42]. The medium for batch and continuous cultivation was Minimal Medium and contained per liter: 4.5 g NH_4_Cl, 1.5 g KH_2_PO_4_, 0.5 KCl, 0.5 MgSO_4_·7H_2_O and 1 ml trace metal solution. 0.01% polypropyleneglycol (PPG 2000, Fluka Chemika) was added to the medium used for continuous cultivation as antifoam agent. The final cell density limiting (growth-limiting) substrate was 7.5 g xylose or maltose (monohydrate) per liter. Carbon sources were heat sterilized separately from the Minimal Medium. During cultivation, temperature was set at 30°C and at pH 3, and kept constant by computer controlled addition of 2 M NaOH. Acidification of the culture broth was used as an indirect measure for growth [43]. Dissolved oxygen tension was always above 40% of air saturation.

Batch cultivation was initiated with inoculation of 4.3 liter minimal medium with 10^9 ^conidia liter^-1^. Germination was induced by addition of 0.003% (w/w) yeast extract. During the first six hours of cultivation the culture was aerated (air flow = 1 l min^-1^) through the headspace of the reactor and stirrer speed kept low at 450 rpm to avoid exhaust of the hydrophobic conidia. After six hours and germination of most conidia (now hydrophilic), air was sparged into the culture broth, mixing was intensified (750 rpm) for more efficient oxygen transfer and 0.01% (v/v) PPG was added.

Xylose was the growth-limiting substrate in the batch culture and during the first five residence times of chemostat cultivation. Continuous cultivation was started after 80 mmol of NaOH had been added to the batch culture (80% of the xylose exhausted or appr. 3.5 g dry weight biomass formed per kg culture). The dilution rate (D) was set to 0.16 h^-1^, which corresponds to 80% and 60% of the maximum specific growth rate (μ_max_) on xylose (≈ 0.20 h^-1^) and maltose (≈ 0.25 h^-1^), respectively. After five residence times (≈ 32 h), the xylose-containing influent medium was replaced with maltose-containing medium. Continuous cultivation was performed for additional five residence times on maltose. Steady-state, a situation where the specific growth rate (μ) is equal to the dilution rate (D), was defined by constant alkali addition rate and constant CO_2_, O_2_, biomass and substrate concentrations after four residence times.

Samples were drawn regularly to monitor culture growth and for determination of steady-state. A special sampling device was used to sample up to 100 ml culture broth in less than a second. The technical principle of the sampling device was previously described [44]. All samples were quickly frozen in liquid nitrogen. Culture filtrate used in substrate and protein determinations was obtained by rapid filtration of culture broth through cotton wool before freezing. 10 mM NaN_3 _was used to conserve samples for determination of organic carbon. Mycelium harvested during steady-state was used in gene expression studies. Mycelium was separated from culture broth by filtration through sintered glass and frozen in liquid nitrogen within 15 sec after sampling.

### Determination of substrate and biomass concentration

Xylose was determined by the modified orcinol reaction described by Standing *et al*. [45]. Residual maltose and glucose (degradation product) was determined enzymatically as glucose, after incubation of culture filtrate with added a-glucosidase (EC 3.2.1.20) [46]. Glucose was determined according to the method of Bergmeyer *et al*. [47] with a slight modification; 250 mM triethanolamine (TEA) was used as buffer (pH 7.5). Dry weight biomass concentration was determined by weighing freeze dried mycelium separated from a known mass of culture broth. Culture broth was filtered through GF/C glass microfibre filter (Whatman).

### Carbon recovery

Influent carbon (continuous culture) was accounted for by carbon-analysis of effluent culture broth (biomass and filtrate) and measurement of CO_2 _in exhaust gas. Biomass carbon was determined according to the method of Kristensen and Andersen [48]. The gas composition of dry mycelium oxidized at 1020°C was determined by gas chromatography with a Carlo Erba 1100EA Elemental Analyzer using acetanilide as standard. Organic carbon in culture filtrate was determined with a TOC-5000 Total Organic Carbon Analyzer (Shimadzu). Malonic acid was used as standard and samples diluted with Millipore water. Content of CO_2 _and O_2 _was measured in exhaust gas of the bioreactor with a Binos 100 M gas analyzer (Rosemount Analyticals).

### Protein determination

Bio-Rad Protein Assay (microassay procedure) was used to determine extracellular protein concentration in culture filtrate. Bovine serum albumin was used as standard.

### RNA isolation and quality control

Total RNA was isolated by modified Trizol extraction. Frozen ground mycelium (≈ 100 mg) was directly suspended in 500 μL Trizol reagent (Invitrogen) and vortexed vigorously for 5–10 min. After centrifugation for 5 min at 13,000 rpm, 450 μL of the supernatant was transferred to a new tube. Chloroform (90 μL) was added and extraction continued as recommended in the Trizol protocol. RNA was purified on NucleoSpin RNA II columns (Machery-Nagel), including a DNaseI digestion step. RNA was eluted in 60 μL miliQ water. RNA quantity and quality was determined on a Nanodrop spectrophotometer and integrity was tested on an Agilent 2100 Bioanalyser.

### Micro-array analysis

Probe synthesis and fragmentation were performed at ServiceXS (Leiden, The Netherlands) according to Affymetrix protocol [49]. DSM (Delft, The Netherlands) proprietary *A. niger *genechips were hybridised, washed, stained and scanned according to Affymetrix protocol [49]. The 3' to 5' signal ratio of probe sets of internal control genes, like *gpdA *(glyceraldehyde-3-phosphatedehydrogenase), *pkiA *(pyruvatekinase), *hxk *(hexokinase) and γ-actin, were below 3 on all arrays (12 arrays). Percentage of probe sets evaluated as present (%P of 14,555 probe sets) was 41 on xylose (6 arrays) and 39 on maltose (6 arrays).

### Normalization, filtering, statistical significance and comparisons

Handling of microarray results and statistical comparisons were performed with GeneSpring (Silicon Genetics, 2004) software. Genes with detection call Present or Marginal in at least one of three replicate measurements were accepted as expressed and used in further analyses. Raw signal values were normalized Per-chip to 50^th ^percentile and Per-gene to median prior to comparison of conditions. Four experimental conditions were compared (maltose- and xylose-limited chemostat cultures of two strains); each condition represented by independent triplicate measurements. Replicate variation of transcript levels was characterized as CV of 25^th^, 50^th^, 75^th ^and 90^th ^percentiles (P) of expressed genes with least variable signal intensities. Xylose-limited cultures (AB94-85/ABGT1026): P25 = 0.08/0.07, P50 = 0.12/0.12, P75 = 0.19/0.18 and P90 = 0.28/0.27. In maltose-limited cultures the corresponding values were 0.13/0.10, 0.19/0.15, 0.27/0.22 and 0.37/0.31. Genes expressed differentially on carbon source were identified by 2-way ANOVA (variances were not assumed equal) with a cut-off p-value of 0.005. Benjamini Hochberg False Discovery Rate [50], FDR, was < 0.05 for genes identified as differentially expressed. Fold change of gene expression from xylose to maltose (maltose/xylose) was calculated for genes with significantly different expression on the two carbon sources.

## Authors' contributions

TRJ carried out chemostat cultivaton, physiological and transcriptomic analyses and wrote the manuscript. TG extracted and purified RNA, carried out initial Northern blot analyses and participated in transcriptomic analysis. AFJR, CvdH and JJLI were involved in writing the manuscript. All authors contributed to design of the experiments and discussion of the results.

All authors read and approved the final manuscript

## Supplementary Material

Additional file 1 **Signal intensities and detection calls.** Transcript profiles of 12 steady states from xylose- or maltose-limited chemostat cultures. Click here for file

Additional file 2 **Steady state transcription of genes in the putative fumonisin gene cluster.** Steady state transcription of genes in the putative fumonisin gene cluster. Click here for file

Additional file 3 **All genes differentially expressed between maltose- and xylose-limited cultures.** 2-way ANOVA applied to isolate effect of carbon source, significance: p < 0.005; 1,247 genes identified as differentially expressed. Click here for file

Additional file 4 **Northern blot analyses.** Northern blot analyses of glaA, bipA and actin expression in xylose- and maltose-limited chemostat cultures. Click here for file

Additional file 5 **Funcat 01.06 Lipid, fatty-acid and isoprenoid metabolism.** Subset of all differentially expressed genes (Additional file 3). Click here for file

Additional file 6 **Funcat 05 Protein synthesis.** Subset of all differentially expressed genes (Additional file 3). Click here for file

Additional file 7 **HacA transcription and processing.** RT-PCR of HacA transcripts from xylose- or maltose-limited steady state cultures of *A. niger *AB94-85 (culture #96) and ABGT1026 (culture #97). Click here for file

Additional file 8 **Funcat 04 Transcription.** Subset of all differentially expressed genes (Additional file  3). Click here for file
