# Supplementary material for: Transcriptomic comparison of Aspergillus niger growing on two different sugars reveals coordinated regulation of the secretory pathway
Source: BMC Genomics. 2009 Jan 23;10:44. doi: 10.1186/1471-2164-10-44 (PMC2639373; doi:10.1186/1471-2164-10-44)
Supplement: Additional file 2 — Steady state transcription of genes in the putative fumonisin gene cluster. Steady state transcription of genes in the putative fumonisin gene cluster. [file 1471-2164-10-44-S2.pdf]

Additional file 2: Steady state transcription of genes in the putative fumonisin gene cluster.

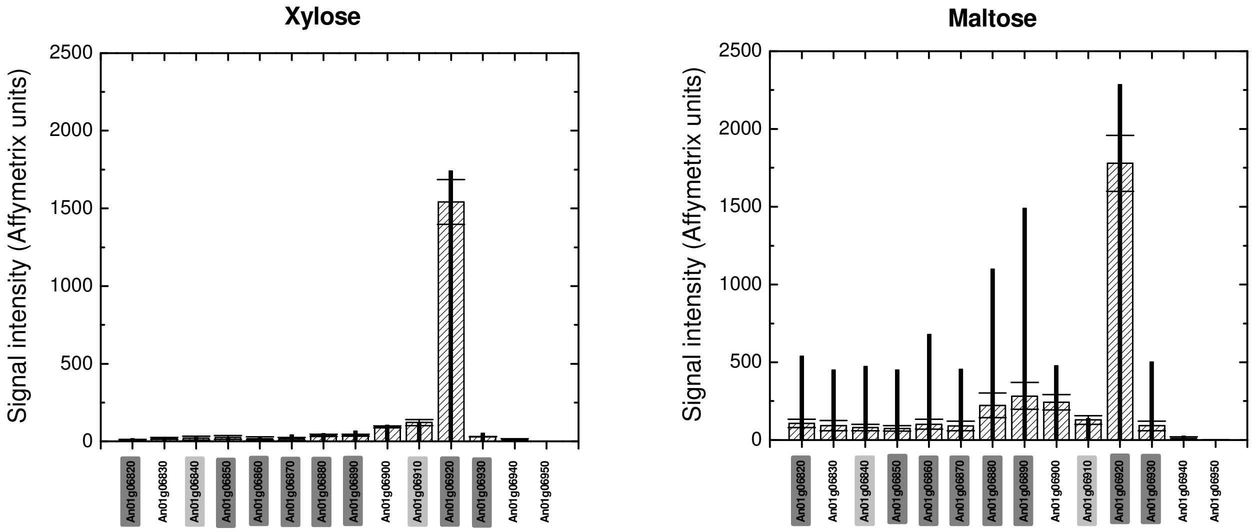

Steady-state transcription profile of genes in the putative fumonisin gene cluster. The mean transcript-level and standard deviation (SD) from 5 independent chemostat cultures and two conditions, xylose and maltose, are shown for the 14 clustered ORFs identified by Pel *et al.* [3]. Solid black bars represent transcription in one chemostat culture (culture ”95”) with remarkably high transcription level. Grey shades mark ORFs homologous to fumonisin genes in *Gibberella moniliformis*. Dark grey shades indicate the eight homologs important for fumonisin biosynthesis in *F. verticillioides*. The fumonisin gene cluster homolgs are listed in the table below.

The fumonisin gene homolog cluster of *A. niger*.

ORF	Description	homolog in *Gibberella moniliformis*
An01g06820	strong similarity to fatty acid omega-hydroxylase (P450foxy) CYP505 –*Fusarium oxysporum*	*fum-6*
An01g06830	similarity to 3-ketosphinganine reductase Tsc10 – *Saccharomyces cerevisiae*	-
An01g06840	strong similarity to acid-CoA ligase Fat2 – *S. cerevisiae*	*fum-10*
An01g06850	similarity to 4-hydroxybutyrate dehydrogenase – *Alcaligenes eutrophus*	*fum-7*
An01g06860	strong similarity to hypothetical protein Fum9 – *G. moniliformis*	*fum-9*
An01g06870	strong similarity to hypothetical protein Fum8 – *G. moniliformis*	*fum-8*
An01g06880	similarity to dihydroflavonol 4-reductase BAA12723.1 – *Rosa* hybrid cultivar	*fum-13*
An01g06890	similarity to peptide synthase PesA – *Metarhizium anisopliae*	*fum-14*
An01g06900	weak similarity to transcription regulator of maltose utilization AmyR – *Aspergillus oryzae*	-
An01g06910	strong similarity to cytochrome P450 CYP94A5 – *Nicotiana tabacum*	*fum-15*
An01g06920	strong similarity to multidrug resistance protein ABCC2 – *Homo sapiens*	***fum-19***
An01g06930	strong similarity to polyketide synthase Fum5 – *G. moniliformis*	*fum-1*
An01g06940	strong similarity to hypothetical transmembrane transport protein SCC30.17c - *Streptomyces coelicolor*	-
An01g06950	strong similarity to polyketide synthase Fum5 – *G. moniliformis*	-

fum-19 = only fum-gene expressed in CBS513.88 under “standard fermentation conditions” – as reported by Pel *et al.* [3].
