# Supplementary material for: Transcriptomic comparison of Aspergillus niger growing on two different sugars reveals coordinated regulation of the secretory pathway
Source: BMC Genomics. 2009 Jan 23;10:44. doi: 10.1186/1471-2164-10-44 (PMC2639373; doi:10.1186/1471-2164-10-44)
Supplement: Additional file 4 — Northern blot analyses. Northern blot analyses of glaA, bipA and actin expression in xylose- and maltose-limited chemostat cultures. [file 1471-2164-10-44-S4.pdf]

Additional file 4. Northern blot analyses.

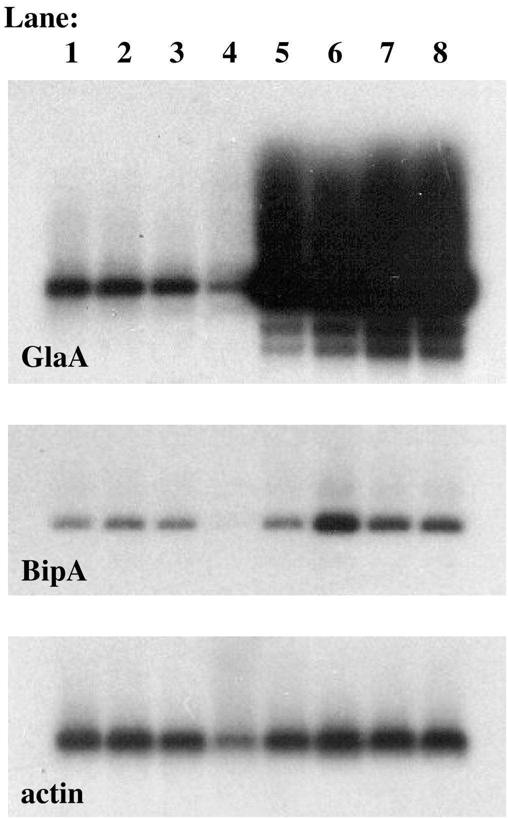

Northern blot analysis of expression of *glaA*, *bipA* and actin in a chemostat culture of *A. niger* (ABGT1026). Lanes: 1, first steady state sample harvested during xylose-limited growth; 2, last steady state sample on xylose; 3, 10 min after medium is changed to maltose-limited; 4, 30 min on maltose; 5, 2 h on maltose; 6. 8 h on maltose; 7, 18 h on maltose; 8, 21 h of growth on maltose (steady state obtained).
