# Supplementary material for: Transcriptomic comparison of Aspergillus niger growing on two different sugars reveals coordinated regulation of the secretory pathway
Source: BMC Genomics. 2009 Jan 23;10:44. doi: 10.1186/1471-2164-10-44 (PMC2639373; doi:10.1186/1471-2164-10-44)
Supplement: Additional file 7 — HacA transcription and processing. RT-PCR of HacA transcripts from xylose- or maltose-limited steady state cultures of A. niger AB94-85 (culture #96) and ABGT1026 (culture #97). [file 1471-2164-10-44-S7.pdf]

Additional file 7. HacA transcription and processing

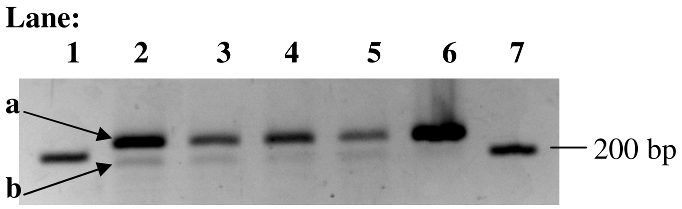

RT-PCR analysis of expression and transcript processing of the UPR transcription factor gene, *hacA*. Lanes: 1, marker; 2, steady state on xylose – AB94-85; 3, steady state on maltose – AB94-85; 4, steady state on xylose, ABGT1026; 5, steady state on maltose, ABGT1026; 6, control - genomic DNA; 7, marker. HacA transcript: a, unspliced; b, spliced.
